# Commentary: Robotic vs. Standard Laparoscopic Technique – What is Better?

**DOI:** 10.3389/fsurg.2015.00038

**Published:** 2015-08-11

**Authors:** Affan Umer, Scott Ellner

**Affiliations:** ^1^Department of Surgery, Saint Francis Hospital and Medical Center, University of Connecticut Health Center, Hartford, CT, USA

**Keywords:** robotic surgery, surgical value, robotic surgery complications, robotic cholecystectomy, selection bias

We read with great interest the recently published article by Ferdinand Kockerling in Frontiers in Surgery (1). The author has provided expert insight into the role of robotic surgery in common abdominal, bariatric, colorectal, and oncologic procedures. The robotic approach allows superiority over the traditional laparoscopic abdominal surgery in terms of a three-dimensional high definition view, seven degrees of freedom of motion, intuitive movements, tremor filtering, and other advantages due to its inherent design (2). Experienced surgeons claim comparable or better outcomes for patients undergoing robotic surgery.

We had recently compared the surgical value, which is defined as the outcome of the procedure divided by the cost to achieve that outcome, of traditional laparoscopic cholecystectomy to the robotic approach. Our outcomes were comparable to national standards in terms of complications, length of stay and readmissions but we became granular with the procedure cost wherein we accounted for supplies, equipment, per use or annual contract costs, and for the operating room (OR) time. Our calculations clearly showed a lower surgical value for the robotic approach. Similarly, concerns for a higher cost have been described for pancreatic surgery (3), colorectal surgery (4), and bariatric surgery (5). Some studies claim a lesser cumulative cost due to a reduction in the hospital length of stay, but at the same time the question arises that how is this reduction in length of stay being achieved if both the laparoscopic and robotic procedures are near similar. Waters et al. (3) reported a shorter average length of stay in their robotic distal pancreatic cohort but that was because of outliers in the laparoscopic group which stayed in excess of 3 weeks. Similarly, the literature is abundant with studies vouching for comparable outcomes but they are plagued with a selection bias for patients with favorable anatomy or a lesser acuity of the disease. This just highlights the dire need to shift from observational data and move toward prospective randomized trials.

Robotic surgery is going through a phase of exponential growth (6). Salisbury et al. (7) commented that structured cross pollination between surgeons and engineers will bridge current deficiencies in robotics. Critical access hospitals may continue to stall on investing due to technology costs, but if this evolution in robotics leads to improved outcomes that argument will be very hard to hold onto. Future improvements expected in robotics aim to miniaturize the console and reduce OR set-up times. These improvements will also include tactile and force sensors to address the lack of haptic feedback. Other advancements are likely to include motion and force scaling for greater precision, and the ability to establish virtual operative boundaries to avoid damaging vital structures. With an industry geared and motivated to redefining surgical norms, my biggest concern is that general surgeons will fall behind the curve and be forced to play catch up. It is critical that adequate education and training keep pace with technology, so the next generation is prepared to recognize and take advantage of the opportunities robotics may provide.

The robotics era is currently catering to the demand for increased patient autonomy but the question remains whether there is sufficient value for critical access hospitals to invest resources in a technology still in its infancy. Training and credentialing remains a big concern and so is the steep learning curve which can potentially introduce a risk for serious injury to patients. This may be part of the reason why penetrance of the robotic approach in visceral surgery has been slow and there has been negligible integration in residency curriculums to introduce the skill early in the surgical career of trainees. Having done this meticulous review, we would like to know the authors view on the future of robotic surgery. We do not think there is evidence in these observational studies that robotic surgery provides enough surgical value. That, however, may change with new innovations in the field. Is it possible that traditional laparoscopic surgeons are resisting the tide of change the same way general surgeons were when laparoscopy was first introduced?

## Conflict of Interest Statement

The authors declare that the research was conducted in the absence of any commercial or financial relationships that could be construed as a potential conflict of interest.
